# Duration Perception and Reading in Typically Developing Adults and Adults with Developmental Dyslexia: Implications for Assessment and Intervention

**DOI:** 10.3390/ejihpe14030046

**Published:** 2024-03-15

**Authors:** Aikaterini Liapi, Susana Silva, Vasiliki Folia

**Affiliations:** 1 Lab of Cognitive Neuroscience, School of Psychology, Aristotle University of Thessaloniki, University Campus, 54124 Thessaloniki, Greece; 2 Center for Psychology, Faculty of Psychology and Educational Sciences, University of Porto, Rua Alfredo Allen s/n, 4200-135 Porto, Portugal

**Keywords:** dyslexia, time perception, beat perception, duration perception, reading skills, entrainment, adults, compensatory strategies

## Abstract

While the link between beat perception and reading skills is attributed to a general improvement in neural entrainment to speech units, duration perception (DP) is primarily linked to a specific aspect of speech perception, specifially discriminating phonemes of varying lengths. Our previous study found a significant correlation between DP and pseudoword reading in both typically developing (TD) individuals and adults with dyslexia (DD). This suggests that, like beat, DP may also enhance overall speech perception. However, our previous study employed a composite measure that did not discriminate speed from accuracy. In this study, we sought to replicate the link between DP and pseudoword reading in a new sample and explore how it might vary depending on the reading parameter being measured. We analyzed the performance of 60 TD vs. 20 DD adults in DP, word reading and pseudoword reading tasks, analyzing the latter for both speed and accuracy. Indeed, duration skills correlated positively with pseudoword reading accuracy. In TD adults, there was no association between DP and reading speed, whereas DD individuals exhibited slower reading speed alongside improved duration skills. We emphasize the potential usefulness of DP tasks in assessment and early intervention and raise new questions about compensatory strategies adopted by DD adults.

## 1. Introduction

Time perception refers to our ability to perceive time distinct from the perception of events (sounds, images) unfolding over time, which is known as temporal processing [1]. Time perception can be divided into two main categories: beat perception, where time is perceived in relation to a regular unit (the beat) [2], and duration perception, where there is no regular underlying reference, and time intervals are judged solely based on their absolute length [3]. Beat-based perception, often referred to as rhythm perception, is pervasive in music, where a typical beat—known as the preferred tempo—lasts around 500/600 ms, or 120/100 beats per minute [4,5]. An example of a beat perception task could be judging whether a series of events follows a regular beat. Estimating the length of a time interval or judging which of two time intervals is longer would be examples of duration perception tasks.

Clinical studies have shown that beat perception difficulties may be present in individuals with dyslexia, a neurodevelopmental disorder characterized by difficulties in reading fluency and accuracy [6]. Individuals with dyslexia exhibit impaired time perception across a diverse range of tasks [7]. Compared to typically developing (TD) controls, children with dyslexia (DD) display increased inaccuracy and variability in beat-related tasks [3], inconsistencies in the neural representation of auditory beats [8], as well as reduced neural synchronization (entrainment) to visually [9] and multimodally presented rhythms [10]. Correlational studies have indicated that beat perception is associated with performance in reading and reading-related tasks, such as pseudoword decoding [11], Rapid Automatized Naming [11,12,13], phonological awareness [14,15], and letter-to-sound knowledge [15], in both TD individuals and those with dyslexia [16,17].

One of the major current explanations for the link between beat perception and reading abilities relies on the role of speech perception in reading. According to the Temporal Sampling Framework (TSF) [18], entrainment to the quasi-regular speech rates—particularly to the stress-related frequencies in the delta range (1–4 Hz, 250–1000 ms)—may be key to acquiring accurate representations of speech units (syllables, phonemes) and, therefore, allowing for accurate grapheme-phoneme correspondences. Despite the imperfect regularity of speech rhythm, a single entrainment ability would subtend both quasi-regular (speech-like) and regular (music-like) auditory input. In line with this, rhythmic training has been shown to enhance speech encoding [19]. Interestingly, the typical musical beat (500–600 ms) also falls within the delta range.

Regarding duration perception, DD participants have shown difficulties in tasks linked to speech-related (very short) durations, such as discriminating onset consonantal duration [15], identifying vowel changes during continuous speech [20], and categorizing phonemes based on length [21]. This suggests that duration perception may be linked to speech encoding through mechanisms other than entrainment, namely the efficient processing of phonemic length. Nevertheless, challenges in discriminating durations within the range of 400–1200 ms, which are longer than those involved in phonemic contrasts, have also been discovered [22]. In a previous study of ours [23], we showed that duration perception for intervals between 134 and 733 ms delimited by beeps was related to pseudoword reading (the number of pseudowords read correctly per time interval) in TD Portuguese adults. This finding would be consistent with duration perception for intervals in the delta range (stress accents in speech) relating to the efficient encoding of speech units in general, thus paralleling beat perception in this regard. In addition, we found no correlation between duration perception for consonants (intervals between 20 and 30 ms) and reading, which strengthens the idea that entrainment to speech rates may be more relevant than perceiving length-based phonemic contrasts in explaining the association between duration perception and reading.

The possibility that duration perception skills for the delta-range intervals benefit reading (including a potential causal relation) is relevant to reading assessment and intervention for more than one reason. On the one hand, duration perception can be assessed from an early age because it depends little on language and much less on print. Early assessment means an increased chance of detecting children at risk for reading difficulties. On the other hand, training duration perception skills in pre- and beginning-schoolers may enhance phonemic representations and, therefore, phoneme-to-grapheme correspondences. Knowing whether this specific timing-reading association is equally present in developmental dyslexia vs. typical development is crucial in this context, given that it informs on the need to consider either similar or different strategies for intervention vs. mere stimulation. In addition to a neurotypical adult sample, Batista et al. [23] examined a complementary sample of adults with dyslexia and found the significant correlation between duration and pseudoword reading to also be present in these individuals. However, Batista et al. [23] used a composite reading measure, referring to both speed and accuracy in reading. Deviations in speed vs. accuracy may have different clinical meanings. For instance, slowed reading may represent a compensatory strategy in dyslexia, while a lack of accuracy will not. Accuracy is also the primary target in both educational and clinical settings. From this viewpoint, associations between duration perception and reading accuracy will be of greater interest in terms of practical applications.

In the present paper, we examined the relation between duration perception and two types of reading measures—speed and accuracy—in a sample of adult readers with vs. without dyslexia. We used the duration perception task from Batista et al. [23] and extracted reading measures for both words and pseudowords. We expected to see significant associations involving pseudowords, but not words, in both groups. Also, we expected speed- vs. accuracy-related reading measures to behave differently across groups concerning links with duration perception.

## 2. Materials and Methods

The study was conducted on a sample of 80 adult native speakers of Greek (60 TD, and 20 with DD). Each group consisted of 6 men (54 women in TD, and 14 women in DD). In the TD group, inclusion criteria encompassed individuals with no neurodevelopmental or other disorders that could affect their cognitive skills. In the case of the dyslexia group, participants were eligible if they had received a formal diagnosis of dyslexia and did not have any comorbid neurodevelopmental or other disorders that could impact their cognitive profile.

All participants were characterized by age, number of years spent in school, and number of years of music training (formal learning and/or informal practice). The latter was considered important because music engages time perception skills. For all three variables, there was no evidence of difference across groups. Participants were also compared for word and pseudoword reading abilities, with the expectation being that individuals with dyslexia would perform more poorly than those without. Table 1 shows that these expectations were met, with DD showing slower reading rates and more errors.

TD participants were enlisted from a university course and offered course credits for their participation. Individuals with dyslexia were recruited using social media posts. Prior to recruitment, participants received an explanation of the study’s procedures and objectives along with information regarding data privacy protocols and participant welfare measures. The study received approval from the Research Ethics Committee of the School of Psychology, Aristotle University of Thessaloniki (Ref. 67β/23-01-2023). All participants gave their informed consent in accordance with the Declaration of Helsinki.

In the duration perception task, participants were asked to compare two time intervals in each of 16 auditory sequences (Table 2). Each sequence consisted of three 50 ms beeps. Beeps 1 and 2 defined the first interval and beeps 2 and 3 the second. Empty (unfilled) intervals were used between the auditory beeps as the basis for duration judgments. Half the sequences featured a longer first interval than the second, creating a perception of acceleration (speed-up). Conversely, the remaining sequences had a longer second interval than the first, resulting in a perception of deceleration (slow-down). Prior to these sequences, a period of silence lasting 200 ms was presented. The mono audio files featured a 16-bit depth and a sampling frequency of 44.1 kHz.

To assess participants’ abilities in word reading and phonemic decoding, the Greek Test of Word Reading Efficiency (TOWRE) [24] was administered. In the first segment of the assessment, participants were required to read a list of 88 words as quickly as possible. The words were divided into four columns, each containing 22 words. For the phonemic decoding segment, participants were asked to read a list of 63 pseudowords as fast as possible. These pseudowords were divided into three columns, with each column containing 21 pseudowords. Prior to the main tests, participants were provided with a short list of words and pseudowords for practice. In each subtest, reading speed was measured by the number of words or pseudowords read within a 45 s timeframe, and reading accuracy was determined by the number of errors made by participants.

Data were gathered remotely. Once participants gave their written informed consent, they received a comprehensive email containing the experiment instructions and a participant code, along with the link for the duration perception experiment. The experiment was created using OSWeb/OpenSesame programming software [25] and was run on a JATOS server [26]. The arrangement of the stimulus sequences was randomized with each iteration of the experiment. In the duration perception task, participants were prompted to evaluate whether three-beep sequences were accelerating (second interval shorter than the first) or decelerating (second interval longer) by pressing either “g”, signifying “γρήγορα” in Greek (meaning “fast”), or “a”, signifying “αργά” in Greek (meaning “slow”). Before the task, a practice trial was presented, and participants moved on at their own pace. The approximate duration of the experiment was 10 min.

Word reading and pseudoword reading subtests were administered separately via Zoom meetings for each participant. During these meetings, the participants’ cameras were disabled, and sessions were not recorded. Access links to these meetings were provided subsequent to the completion of the experimental tasks at a predetermined time. Six participants from the TD group did not proceed with this part. Zoom sessions lasted an average of approximately 10 min for TD individuals and around 15 min for participants with dyslexia.

All statistical analyses were performed using the JASP 0.17.3.0 software [27]. Non-parametric alternatives were employed when the data did not meet parametric assumptions (normality and equality of variances). Due to our limited sample size, we opted for more robust hypothesis testing through Bayesian analyses. Bayes Factors (BFs) were used to quantify the strength of evidence in support of the alternative hypothesis (H1) against the null hypothesis (H0). Following the guidelines proposed by van Doorn et al. [28], BFs ranging between 1 and 3 were considered indicative of weak evidence, from 3 to 10 as moderate, while BFs in the range of 10−30 were regarded as strong support for H1 and values above 30 indicated very strong support. Conversely, BFs below 1 favored H0, with values between 1 and 0.33 indicating weak evidence, from 0.33 to 0.10 suggesting moderate evidence, and 0.10−0.03 signifying strong evidence in favor of H0. BFs below 0.03 provided very strong evidence supporting H0.

To cross-check the diagnoses of participants in the dyslexia group, we compared the two groups’ performance on reading measures using the Bayesian Mann–Whitney independent samples test (as described above in the participants’ section). We also conducted the same test to determine whether participants with dyslexia performed worse than TD individuals in the duration perception task (accuracy scores on a scale from 0 to 100). Subsequently, we aimed to determine whether any of the demographic variables (age, education level, music education/practice, gender) were related to the study variables (duration perception and reading measures). This assessment was conducted to decide if any demographic variables should be included as covariates in subsequent analyses. To that end, we ran Bayesian Kendall’s tau correlations, including the continuous variables (age, schooling, music practice), and conducted Bayesian Mann–Whitney independent samples tests for comparison on the categorical variable of gender.

To examine the relationship between duration perception and reading, we conducted four Bayesian linear regression analyses, one for each reading measure as the dependent variable, with population and duration perception as predictors. First, the competing models (one vs. two predictors, with vs. without an interaction term) were ranked using the Bayesian regression analysis based on their degree of plausibility (support against the null model) and their fir (percentage of the variance explained). Then, we assessed the inclusion BFs of the posterior coefficients and the inclusion probabilities graph provided by the analyses. A predictor was considered for inclusion when the inclusion BFs were greater than 1 and supported by the related inclusion probabilities graph. In cases where the inclusion of the interaction predictor was supported, separate Bayesian Pearson’s r and Kendall’s tau correlation analyses were conducted between duration perception and the reading measure for the TD and dyslexia groups separately for clarification. The BFs and BF robustness graph were checked as described above.

## 3. Results

**Figure 1:**
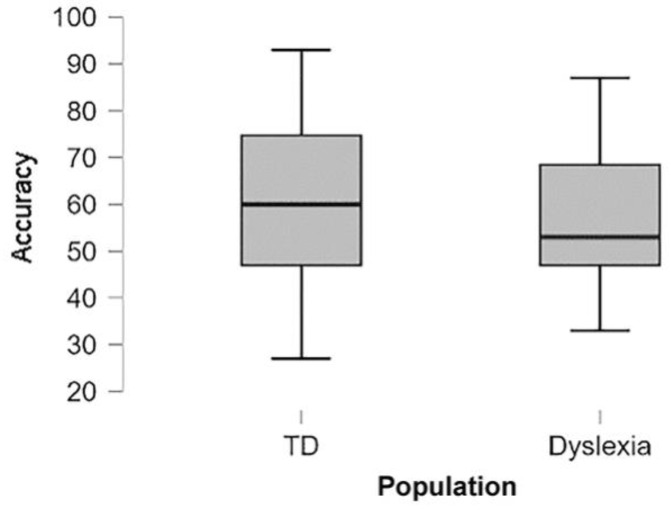
Accuracy (0–100) in the duration perception task for the typically developing (TD) vs. dyslexia group.

Figure 1 presents duration perception accuracy values per group. The median accuracy score of individuals with dyslexia was lower than that of controls. However, the Bayesian Mann–Whitney analysis (W = 654) showed moderate support for the null hypothesis (BF = 0.279), thus suggesting the absence of significant cross-group differences in duration perception.

As shown in Table 3, there was no relevant association between sociodemographic and the study variables (duration perception and/or reading). Therefore, we did not include any of the sociodemographic variables as control factors in the main analysis.

The main analysis showed that both reading speed and reading accuracy for pseudowords (number of errors: fewer errors, increased accuracy) were significantly predicted by duration perception, population, and their interaction (Table 4). The follow-up Bayesian correlations provided near-to-moderate support (BF = 2.982) for a strong [29] negative correlation between duration perception and reading speed in the dyslexia group. Simultaneously, support was provided, albeit weakly, for a negative correlation between duration perception and the number of errors in the TD group. This means that TD individuals with better duration perception skills were more accurate in pseudoword reading than individuals with poorer timing performance. Though the DD group showed a similar pattern, the results did not survive the endorsement criteria we established (BF < 1). No significant associations were found between duration perception and word reading, with the best model consisting of population as a single predictor.

## 4. Discussion

In the current investigation, we aimed to gain a better understanding of the positive association we found in a previous study between duration perception for intervals ranging between 134 and 733 ms and a measure of pseudoword reading that combined speed and accuracy [23] in both adults with and without dyslexia. Specifically, we wanted to determine (1) whether this association would replicate in new groups of adults and, if so, (2) how it could depend on the reading parameter being measured (speed vs. accuracy). To that end, we analyzed the performance of 60 typically developing adults vs. 20 individuals with dyslexia in duration perception, word reading, and pseudoword reading tasks.

Concerning (1), duration skills correlated positively with pseudoword but not with word reading, as observed in Batista et al. [23]. Both participants with and without dyslexia showed positive associations with reading accuracy (a negative correlation with number of errors), even though only the correlation in the control group was backed by Bayesian evidence. Pseudoword reading, a task during which readers cannot employ automatized word form recognition, relies on the robustness of the available combinations of possible grapheme-phoneme representations. These, in turn, may benefit from duration perception skills that facilitate phonemic encoding.

Regarding (2), results for reading speed differed from those for accuracy. While neurotypical adults showed no association between duration perception and reading speed (i.e., the number of pseudowords read correctly or not), those with dyslexia exhibited a negative correlation, indicating that better duration skills go along with slower reading in this group. This may have occurred because participants with dyslexia who possessed better duration skills might have experienced fewer reading difficulties, thus (unlike those ‘who struggled’) finding it worthwhile to use compensatory strategies. In summary, individuals with dyslexia and better duration skills may have intentionally slowed down to enhance accuracy, because they knew it would be compensatory, while others may not have attempted to do so. As for control participants, compensation was likely unnecessary. An alternative explanation could be that duration skills determined participants’ ability to implement the compensatory strategy, i.e., to control and monitor the speed at which they read. In other words, dyslexics with better duration skills may have been more capable of monitoring their own reading speed, a skill necessary to trigger compensatory strategies.

Factors such as attention, and the nature of the task (attentive or unattentive) are also crucial when analyzing results related to time perception tasks and might explain some inconsistencies in the results in our paper. For example, it is possible that unconscious mechanisms related to timing and the integration of auditory and visual information not examined by our study also come into play. Casini et al. [22], have highlighted that individuals with dyslexia may exhibit greater perceptual variability in auditory and visual temporal tasks, but not in tasks assessing other skills, such as intensity perception, questioning attention lapses as the primary mechanism. Notably, their study employed both explicit and implicit temporal processing tasks. In contrast, our study exclusively relied on explicit tasks for assessing beat and duration perception, potentially overlooking unconscious temporal processing or links to the inattentive use of contextual cues during reading. Based on the above, a more comprehensive approach to studying the suggested correlations could involve the inclusion of tasks that assess temporal skills in both explicit and implicit ways, and, as such, the possible interplay between implicit and explicit processes could effectively be illuminated.

Concerning practical implications, the fact that reading accuracy—the foundation of literacy—benefited from duration perception skills further highlights the importance of timing skills in clinical practice. Typically developing individuals showed a stronger association than those with dyslexia, but the profile was similar across groups, and the sample size may have contributed to the weaker results in the dyslexia group. In this sense, assessing and promoting duration skills before and during learning to read is a possibility that, in our opinion, should not be discarded. As for reading speed, our results did not indicate that duration skills may enhance it. However, they may still be important, in that they help to set non-trivial expectations for individuals with dyslexia who possess better duration skills. At least in adults, improved duration perception seems to predict slower reading, but this slower reading may be a positive sign—an indication that compensatory strategies are at play.

While the main results of this study did not contradict our expectations, they do contain some intriguing details that may warrant future attention. The first concerns the rhythm characteristics of the Greek language, which could have potentially hindered our attempt to replicate Batista et al.’s [23] findings but did not. Our initial interpretation of Batista et al.’s findings [23] was that the early ability of Portuguese participants to perceive durations in the stress-related range (around 500 ms) could have been responsible for efficient speech encoding through entrainment to stress rates, which are relevant and distinct in European Portuguese [30]. Phonological representations encompass units smaller than inter-stress intervals, such as syllables or phonemes, but inter-stress intervals, at least when they are relevant for a given language, may serve as the basic structure upon which these smaller units are acquired as speech perception develops [18]. Syllables, in turn, may serve as the framework for phoneme representations [31]. Unlike Portuguese, where syllable-timing is prevalent, Greek leans towards stress-timing [32]. Therefore, the question arises: why would entrainment to stress rates enhance speech encoding in Greek participants? One possibility is that the duration task we used might have also captured syllable-related lengths. We have referred to the time intervals in our task as stress-like (250–1000 Hz, 1–4 Hz, delta frequencies), but they also include smaller intervals (134 to 233 ms, constituting 25% of all intervals) representing syllabic rates. In summary, Greek speakers with better duration skills as measured in our task might have also been more efficient entrainers to syllabic structure during language acquisition, resulting in more robust phonological representations. To further test this hypothesis, increased control over interval lengths (e.g., separating theta from delta range) would be necessary.

A second relatively unexpected detail was the lack of evidence for cross-group differences in duration perception skills. Although these differences were not a prerequisite for examining the relation between duration perception and reading speed/accuracy in the two groups (unlike differences in reading skills), we expected participants with dyslexia to perform worse than controls. One possibility is that duration perception difficulties, though potentially detrimental to the acquisition of phonemic representations, mostly affect those of a younger age. This is supported by some studies with adults that have provided null effects [33]. While compensated adults may have overcome such low-level deficits to some extent, any early deviation in duration perception could have played a significant role in earlier development stages [20], particularly during early speech encoding and phoneme-grapheme conversion. The ideal way to test this would be to implement a cohort study measuring both duration perception and pseudoword reading across development. A more feasible alternative would be to start with cross-sectional studies comparing children and adults.

Beyond these emerging questions, we should highlight at least two limitations of the current study that may also warrant future research attention. One limitation is that duration perception results might have been influenced by factors such as memory, attention, and other cognitive abilities [34], as time intervals require continuous monitoring of their starting and closing points. In this context, it is also possible that decision-making abilities may have played a role, along with participants’ experience of task demands (e.g., working memory overload) [35]. Evaluating participants for these domain-general abilities through detailed cognitive assessments and subsequently controlling for them (if necessary) would be a way to improve in this area. The other limitation concerns the relatively speculative nature of the explanations based on compensatory strategies we have proposed. Although they make sense, we lack empirical evidence that they were present, and even less so that they were responsible for the negative correlation between duration perception and reading speed. Some of these compensatory strategies may have originated from the quality of educational interventions received during childhood. In Greece, both presently and two decades ago (given that our participants are currently university students), initiatives aimed at providing support and dyslexia training programs have been undertaken, although their implementations nationwide have not always been consistent or uniform. Therefore, it is important to work on a more elaborate study design that could actively measure and/or control for such individualized compensatory strategies.

## 5. Conclusions

Despite its limitations, our study has strengthened the available evidence that duration (not just beat) perception, may be related to phonemic encoding beyond phoneme length, possibly through entrainment to relevant speech rates. On the practical side, we have reinforced the idea that duration perception skills may facilitate the early stages of reading with regard to letter-to-sound correspondences. Furthermore, our findings suggest that it is reading accuracy, the primary target of intervention, and not reading speed, that may benefit from duration perception skills. This holds true in typical development and potentially in dyslexia as well.

## Figures and Tables

**Table 1:** Sociodemographic (values in years) and reading-related characteristics of the typical development (TD) vs. developmental dyslexia (DD) groups.

Descriptives Mean ± Standard Deviation			Comparison		
	TD *n* = 60 Male = 6	DD *n* = 20 Male = 6	BF ^2^	W ^2^	Rank-Biserial Correlation ^2^
Age	20.450 ± 1.016	21.400 ± 2.798	0.289	530.500	−0.116
Schooling	14.333 ± 0.655	14.800 ± 2.167	0.345	502.000	−0.163
Music training	2.142 ± 3.442	1.000 ± 1.974	0.338	695.000	0.158
Word reading					
Speed ^1^	81.444 ± 8.388	67.100 ± 10.794	47.419 ****	929.500	0.721
Errors ^1^	0.037 ± 0.191	1.050 ± 1.572	3.865 *	286.000	−0.470
Pseudoword reading					
Speed ^1^	58.833 ± 4.334	48.550 ± 7.258	69.021 ****	965.000	0.787
Errors ^1^	1.296 ± 1.644	4.900 ± 4.241	8.720 **	221.000	−0.591

^1^ Speed was measured by counting the number of words/pseudowords read in 45 s, and accuracy was assessed based on the number of errors. ^2^ Comparisons were conducted using Bayesian methods (BF = Bayes Factor; W = Bayesian; Mann–Whitney test; Rank-biserial correlation indicates effect size; the asterisks indicate evidence in favor of the alternative hypothesis, with more asterisks indicating stronger evidence). For more details, please refer to the statistical analysis section.

**Table 2:** Time structure of beep sequences used in the duration perception task (ms).

Type	Interval 1	Interval 2	Difference	Type	Interval 1	Interval 2	Difference
Slow down	300	433	−133	Speed up	433	300	133
Slow down	167	300	−133	Speed up	300	167	133
Slow down	433	467	−34	Speed up	467	433	34
Slow down	167	733	−566	Speed up	733	167	566
Slow down	300	467	−167	Speed up	467	300	167
Slow down	134	434	−301	Speed up	433	134	299
Slow down	233	534	−301	Speed up	534	233	301
Slow down	433	500	−67	Speed up	500	433	67

**Table 3:** Correlations between sociodemographic and study variables (BF = Bayes Factor).

	Age	Schooling	Music Training	Gender
	Kendall’s tau (BF)			Mann–Whitney (BF)
Duration perception	−0.034 (0.160)	−0.013 (0.148)	0.128 (0.587)	0.050 (0.286)
Word reading				
Speed ^1^	0.110 (0.392)	0.081 (0.253)	0.094 (0.301)	−0.142 (0.332)
Errors ^1^	−0.033 (0.165)	−0.049 (0.182)	−0.036 (0.167)	0.216 (0.407)
Pseudoword reading				
Speed ^1^	−0.112 (0.407)	−0.133 (0.606)	0.065 (0.212)	0.128 (0.305)
Errors ^1^	0.097 (0.317)	0.118 (0.452)	−0.108 (0.376)	0.051 (0.301)

^1^ Speed was measured by counting the number of words/pseudowords read in 45 s, and accuracy was assessed based on the number of errors.

**Table 4:** Predictors of reading performance (TD = typical development; BF = Bayes Factor).

Dependent Variable	Best Bayesian Regression Model ^1^	Bayesian Correlations Duration-Reading (Kendall’s Tau)	
		TD	Dyslexia
Word Reading	Population (R^2^ = 33.6%)		
Pseudoword Reading	Population (R^2^ = 23.5%)		
Word Reading	Full model (R^2^ = 50.8%)	τ_b = −0.034 BF = 0.189	r = −0.502 BF = 2.982 *
Pseudoword Reading	Full model (R^2^ = 34.9%)	τ_b = −0.207 BF = 1.924 *	τ_b = −0.245 BF = 0.838

* Weak evidence for the alternative hypothesis (1 < BF < 3). ^1^ Best model chosen among (1) Population, (2) Duration perception, (3) main effects of both, or (4) the full model with main effects and their interaction.

## Data Availability

The dataset generated in this study is available at the OSF link osf.io/23er4.
